# High proportion of overt diabetes mellitus in pregnancy and missed opportunity for early detection of diabetes at a tertiary care centre in Pakistan

**DOI:** 10.12669/pjms.36.ICON-Suppl.1723

**Published:** 2020-01

**Authors:** Aisha Syed Wali, Raheela Rafique, Sundus Iftikhar, Rakhshinda Ambreen, Mohammad Yawar Yakoob

**Affiliations:** 1 Aisha Syed Wali, FCPS. Sheikh Saeed Memorial Hospital, Indus Health Network, Karachi, Pakistan; 2 Raheela Rafique, MBBS. Sheikh Saeed Memorial Hospital, Indus Health Network, Karachi, Pakistan; 3 Sundus Iftikhar, MS. Indus Hospital Research Center, Indus Health Network, Karachi, Pakistan; 4 Rakhshinda Ambreen, BDS. Indus Hospital Research Center, Indus Health Network, Karachi, Pakistan; 5 Mohammad Yawar Yakoob, DS. Indus Hospital Research Center, Indus Health Network, Karachi, Pakistan

**Keywords:** Diabetes mellitus in pregnancy, Gestational diabetes, Overt diabetes in pregnancy, Pre-gestational diabetes

## Abstract

**Objectives:**

To determine the frequency of diabetes in pregnancy (DIP), namely pre-gestational, gestational (GDM) and overt diabetes mellitus (DM) in women registered for delivery.

**Methods:**

A retrospective chart review of antenatal women registered between January 01 to August 31, 2017 was performed. Gestational age, diagnosis of DIP, glucose levels at diagnosis and other relevant data was extracted. The effect of various fasting blood glucose (FBG) thresholds for diagnosis of DIP was assessed.

**Results:**

DIP was diagnosed in 21.8% women (pre-gestational: 2%, GDM: 81.2%, overt: DM: 16.8%). In early registrants, 30.2% were detected through screening. However, 55.3% of women registered late. Women with pre-gestational DM were older, had more miscarriages, and greater personal and family history of diabetes versus GDM and overt DM. Raising the diagnostic threshold of FBG from 92 mg/dl to 95 mg/dl missed three women (0.1%) and to 105 mg/dl, missed six women (0.2%).

**Conclusion:**

We observed a high proportion of overt DM. In early registrants, almost one third of DIP was diagnosed in the first half of pregnancy, an opportunity missed in late registrants. Altering diagnostic thresholds of DIP affected only a small proportion of women.

## INTRODUCTION

Diabetes Mellitus (DM) is a major global health emergency of the 21st century.^1^ Diabetes in pregnancy (DIP) is also increasing along with other forms of diabetes mellitus. DIP can be classified into three types: (a) gestational diabetes mellitus (GDM), that develops during pregnancy; (b) overt DM, undiagnosed pre-existing diabetes mellitus that is detected in pregnancy; and (c) pre-gestational DM, seen in women with established diabetes who become pregnant. About 16.2% of live births are exposed to hyperglycemia in utero, 85.1% of which is due to GDM.^2^ The prevalence of GDM is highest in Southeast Asia, mostly in low or middle-income groups, estimated at approximately 24.2%.^2^

DIP not only adversely affects the fetus and the neonate, but also has long-term effects, including childhood obesity, metabolic syndrome and diabetes mellitus in adult life.^1^-^3^ This increases the disease burden. Good glycemic control is associated with better perinatal outcomes.^4^,^5^ The threshold of fasting plasma glucose (FPG) for the diagnosis of GDM has changed over the years and expecting better neonatal outcomes, the FPG criteria has lowered.^1^ Now in most of the institutions diagnosis of GDM is based on FPG of 92 mg/dl^6^-^9^ on recommendations of the “International Association of Diabetes and Pregnancy Study Groups” (IADPSG) 2010 criteria.^10^

At The Indus Hospital (TIH) Karachi, the IADPSG 2010 criteria^10^ is followed for screening and diagnosis of diabetes in pregnancy. As such, it is based on FBG, glycated hemoglobin (HbA1c) or a 2-hour 75 g oral glucose tolerance test (OGTT) according to gestational age and risk factors. We conducted this study to determine the burden and frequency of GDM, overt DM and pre-gestational DM amongst population that we register at TIH. We also compared the difference between early and late screened patients. Finally, we compared the frequency of GDM at different thresholds of FPG i.e. 105 mg/dl, 95 mg/dl and 92 mg/dl.

## METHODS

A retrospective observational study was conducted at TIH, a tertiary care centre located in Korangi, Karachi, Pakistan. This hospital provides free-of-cost quality care to underprivileged patients. Electronic medical records of women registered for delivery from January 1, 2017 to August 31, 2017 and who were less than 34 weeks by the end of study period, were retrieved from Hospital Management Information System (HMIS). Data was collected manually on forms designed for the study. It included demographic data, DM screening results, gestational age at booking and screening. All necessary ethical approvals were in place (IRD_IRB_2017_10_003).

Women with known diagnoses with type 1 or type 2 diabetes at the time of registration were labeled “pre-gestational DM”. All other registered pregnant women were screened. At the first pre-natal visit at less than 24 weeks gestation, FPG was used as early screening and diagnostic tool of DIP. Glycated hemoglobin (HbA1c) was additionally checked in high-risk groups (i.e. diabetes in first degree relative, previous history of GDM, body mass index > 30 kg/m^2^, known polycystic ovarian syndrome (PCOS), previous history of macrosomia, unexplained stillbirth or neonatal death).^10^ As per protocol, women with PCOS on metformin, stopped this drug two weeks prior to taking the screening test. In accordance with IADPSG criteria,^10^ women were categorized into three groups. Those with FPG < 92 mg/dl were labeled non-diabetic; those with FPG 92 mg/dl – 125 mg/dl as GDM and those with FPG ≥ 126 mg/dl and/or an HbA1c of ≥ 6.5% as overt DM. Women presenting for registration at 24 weeks’ gestation or later along with those who tested negative at early screening were screened at 24-28 weeks’ gestation with a 2 hour 75-g OGTT. Using OGTT, any one or more of the following criteria met at any time in pregnancy is diagnostic of DIP; FPG ≥ 92 mg/dl, 1-hour glucose ≥ 180 mg/dl, 2-hour glucose ≥ 153 mg/dl.^10^ Women not tolerating OGTT were screened with 50 g Oral Glucose Challenge Test (GCT); with a threshold for GDM being 1-hour glucose level ≥ 140 mg/dl.^11^

All pregnant women with diagnosed pre-gestational type 1 and type 2 diabetes mellitus at the time of registration and those diagnosed as GDM or overt DM on the basis of the above-mentioned screening were included in the sample. Women with secondary diabetes mellitus and those on steroid therapy were excluded. Outcomes measures were frequency of DIP, including frequency of pre-gestational DM, GDM and overt DM. The detection of GDM and overt DM on early and late screening was analyzed. The frequency of GDM detected using FPG ≥ 92mg/dl was also compared with frequency on of GDM using FPG ≥ 95mg/dl and FPG ≥ 105mg/dl as diagnostic thresholds.

Data was entered and analysed using SPSS version 21.0. A descriptive analysis was conducted for quantitative variables after assessment for normality. Normally distributed variables were reported as mean ± standard deviation and non-normal variables were reported as median (with Inter Quartile Range, IQR). The frequency and percentage of qualitative variables was assessed. Student t-test was used to compare continuous normal variables, Mann-Whitney U-test for continuous non-normal variables and Chi-square or Fisher’s exact test was used for categorical variables. All p-values were two-sided and considered statistically significant if less than 0.05.

## RESULTS

A total of 2462 women were registered during the study period of eight months. A diagnosis of DIP was made in 537 (21.8%). The frequency of pre-gestational DM was 0.5% (n=11/2462); among whom, one woman had Type-1 and ten had Type-2 DM. The remaining 2451 were screened for DM and 526 (21.5%) were positive. Of these, 17.8% (436/2451) had GDM and 3.7% (90/2451) had overt DM. Of all women with DIP (n=537), overt DM was seen in 16.8% (n=90), GDM in 81.2% (n=436) and remaining 2% (n=11) were pre-gestational.

**Fig.1:**
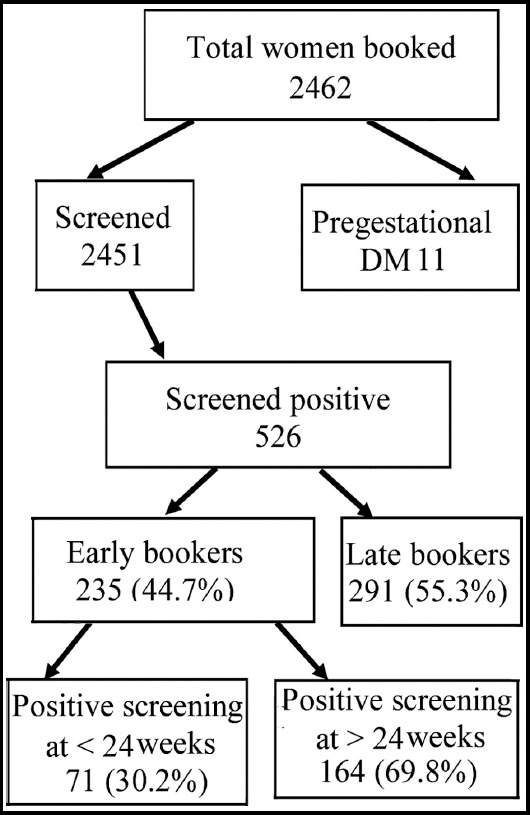
Flow-diagram showing the diagnosis of diabetes in pregnancy with early vs. late booking.

Of those who were screened positive for DIP, 44.7% women (n=235) were early bookers, i.e. registered within 24 weeks gestation. Of these, 30.2% (n=71/235) were diagnosed with DIP on initial screen, while the remaining 69.8% (n=164) were screened negative at early screening for DIP but found to be positive on later screen at 24-28 weeks gestation (Fig.1).

The distribution of demographics and risk factors across categories of DIP is shown in Table-I. Women with pre-existing DM were older compared to women with GDM and overt DM. They had a history of two or more miscarriages, GDM in the past and a family history of diabetes compared to those with GDM and overt DM (p < 0.05).

**Table I:** The distribution of demographics and risk factors across categories of DIP.

	Pre-gestational DM n (%)	GDM n (%)	Overt DM n (%)	P-value
Age in years (median)	32 (30-35)	26 (23-30)	28 (25-31.5)	<0.001**
Gestational age in weeks median (IQR)				
At booking	23 (12-26)	24 (20-27)	24.5 (21-26)	0.465
At diagnosis	-	26 (24-28)	25.5 (24-27.25)	0.185
Education level				
Illiterate	0 (0)	48 (11.4)	11 (12.2)	0.834
Below matric	4 (50.0)	126 (29.8)	155 (29.8)	
Matric	3 (37.5)	144 (34.1)	31 (34.4)	
Intermediate	2 (25)	68 (16.1)	12 (13.3)	
Graduate	2 (25)	30 (7.1)	8 (8.9)	
Post-graduate	0 (0)	6 (1.4)	0 (0)	
Substance use				
None	11 (100)	377 (88.1)	81 (94.2)	0.249
Cigarette smoking	0 (0)	1 (0.2)	0 (0)	
Betel nuts	0 (0)	48 (11.2)	5 (5.8)	
Chewable tobacco	0 (0)	2 (0.5)	0 (0)	
Parity				
Nullipara	1 (9.1)	154 (35.4)	23 (25.6)	0.107
Primigravida	3 (27.3)	130 (29.9)	30 (33.3)	
Multigravida	7 (63.6)	151 (34.7)	37 (41.1)	
Past history of miscarriages				
Overall miscarriage	4 (36.4)	105 (24.1)	27 (30)	0.371
≥ 2 miscarriages	3 (27.3)	21 (4.8)	6 (6.7)	<0.01**
Past history of premature delivery (< 37 weeks)	1 (9.1)	3 (0.7)	1 (1.1)	0.088
Past history of low birth weight (< 2.5 kg)	0 (0)	0 (0)	1 (1.1)	0.188
Past history of birth weight > 3.5 kg	0 (0)	2 (0.5)	0 (0)	1.00
Past history of congenital anomaly	0 (0)	3 (0.7)	0 (0)	1.00
Past history of intra-uterine/neonatal death	0 (0)	28 (6.4)	5 (5.6)	0.66
Past history of GDM	3 (27.3)	7 (1.6)	1 (1.1)	0.001**
Past history of pregnancy-induced hypertension	0 (0)	14 (3.2)	3 (3.3)	1.00
Past history of pre-eclampsia	0 (0)	8 (1.8)	1 (1.1)	1.00
Past history of C-section	4 (36.4)	94 (21.6)	25 (27.8)	0.248
Family history of diabetes	6 (54.5)	91 (21.1)	32 (35.6)	0.001**
Family history of hypertension	2 (18.2)	86 (20)	11 (12.5)	0.263
Family history of pre-eclampsia	0 (0)	1 (0.2)	1 (1.1)	0.342

** P value <0.05

Applying the threshold of FPG to 95 mg/dl, three cases of GDM (0.1%) would have been missed while applying a threshold of 105 mg/dl, 6 cases of GDM (0.2%) would have been missed in 2451 women screened (Table-II).

**Table II:** Diagnosis of DIP at variable diagnostic criteria of fasting plasma glucose (FPG) in mg/dl.

	Cut-off FPG ≥ 92 mg/dl	Cut-off FPG ≥ 95 mg/dl	Cut-off FPG ≥ 105 mg/dl
Number of non-diabetic women	0	3	6
Number of women with GDM	436	433	431

## DISCUSSION

Our study set out to determine the burden of diabetes in the pregnant population we serve. Our overall finding of 21.8% of registered pregnant women suffering from DIP correlates with prevalence in Southeast Asian population estimated at 24.2%.^2^ The proportion of GDM among the women suffering from DIP was 81.2% and is consistent with the global proportion of GDM cited as 86.4% of total glucose intolerance in pregnancy.^2^ It must be emphasized that 16.8% of women had overt diabetes mellitus in our screening program - a large proportion of young women unaware of their disease. Comparatively, 7-8% of women worldwide have overt DM when screened in this way.^2^ Our results may reflect a lack of health awareness or education in our patient population. There may be poor access to primary health care in low-income settings as seen in the communities served by TIH. The high prevalence of DIP strongly warrants advocacy for primary and secondary prevention of diabetes, as recommended by the International Diabetes Federation.^2^

The frequency of GDM (17.7%) in our study is comparable to national data from Pakistan including Bahawalpur in 2012 (19.0%)^12^ and the Karachi/Hyderabad region in 2013-2016 (11.8%).^13^ Southeast Asian countries such as Vietnam (20.1%),^14^ Singapore (18.9%),^14^ China (11.9%)^14^ and Malaysia (11.8%)^14^ also report similar figures. However, other regional neighbors have reported variable results, depending on the diagnostic criteria used. A study from India^15^ reported prevalence to be as low as 7.1% based on American Diabetes Association criteria. Similarly, in Nepal,^16^ WHO and IADPSG criteria for diagnosis was compared and prevalence of 2.5% and 6.6% respectively were found. However, a study from Bangladesh^17^ reported GDM as 36% according to WHO-1999 criteria.

We observed that 55.3% of women diagnosed with DIP registered after 24 weeks of gestation. In women who registered earlier than 24 weeks of gestation, 30.2% of women were diagnosed with DIP on early screening. A large proportion of late registrants may have missed this opportunity for detection and control DIP earlier in pregnancy. Larger prospective studies are required to infer the proportion of DIP detected in early pregnancy, however early detection is important to prevent in utero exposure to hyperglycemia. This further emphasizes the need for advocacy for early registration and screening for diabetes in our population to enable secondary prevention of DIP. The high frequency of DIP and its rising trend highlights the need for primary prevention at the community level as well, promoting lifestyle modifications and healthy eating habits in young girls.

Over recent decades, there has been tightening of the diagnostic criteria for GDM in an attempt to improve neonatal outcomes. World Health Organization considered FPG ≥ 140 mg/dl in 1999, FPG ≥ 126 mg/dl in 2006,^18^ NICE guideline recommended FPG ≥105mg/dl in 2008,^19^ and the IADPSG^20^ consensus was set at FPG ≥ 95 mg/dl as the diagnostic criteria for GDM but further lowered to diagnostic cut off to ≥92 mg/dl in 2010^10^ and now most institutions, including those mentioned above and ours, have adopted this. However studies are emerging that question the benefit of lowering the FPG with respect to fetal outcomes as costs of over-diagnosis and over-medication can burden health systems, in addition to increasing anxiety.^21^-^25^ Comparing our frequency of GDM based on IADPSG 2010 criteria with those of different thresholds of FPG, we found relatively small numbers of women being affected. There is a need for larger studies to define the diagnostic criteria for GDM that best correlates with good neonatal outcomes in the South-East Asian population while balancing cost and burden on healthcare.

## CONCLUSION

We observed a high frequency of DIP with a higher proportion of overt DM. About one third of cases of DIP were diagnosed in the first half of pregnancy, an opportunity that was missed in women who registered late. The effect of varying diagnostic criteria on frequency of GDM was small and warrants further studies to assess its significance.
